# Postpartum automated estrus alert phenotypes in seasonal-calving, pasture-based dairy cows

**DOI:** 10.3168/jdsc.2025-0921

**Published:** 2026-02-19

**Authors:** E.M. Sitko, J. O'Connor, J. McCarthy, S.T. Butler

**Affiliations:** 1 Teagasc, Animal and Grassland Research and Innovation Center, Moorepark, Fermoy, Co. Cork, P61 C996 Ireland; 2 MSD Animal Health, Red Oak, Leopardstown, Dublin, D18 X5K7 Ireland; 3 Irish Cattle Breeding Federation, Ballincollig, Co. Cork, P31 D452, Ireland

## Abstract

The objectives of this retrospective observational study were to (1) characterize postpartum estrus phenotypes in spring-calving, pasture-based dairy cows; (2) evaluate associations between cow-level factors and the timing of return to estrus; and (3) evaluate associations between pre-breeding estrus phenotypes and first-service pregnancy per artificial insemination (P/AI). Individual cow data were available from 4,586 lactations across 19 herds in 2022 and 2023. All cows were monitored with a neck-mounted activity monitoring device (SenseHub, MSD Animal Health). The first automated estrus alert occurred on 34 ± 16 d postpartum (mean ± SD); 25%, 51%, and 24% of cows were classified as early (≤21 d), intermediate (22–42 d), or late (>42 d), respectively. First-lactation cows had a lower hazard of first estrus compared with multiparous cows. Cows in the medium and high tertiles for genetic merit for fertility traits had a greater hazard of first estrus and achieved greater first-service P/AI compared with cows in the low genetic merit for fertility tertile. Only a small proportion (2%) of interestrus intervals were between 2 and 7 d. Most interestrus intervals were normal length (18–24 d), although short (8–17 d) and prolonged (>24 d) intervals were more common after the first estrus compared with subsequent estrus events. Cows that expressed at least one estrus before first service had greater P/AI (57% vs. 47%), and cows inseminated following a normal interestrus interval had greater P/AI (58%) than those inseminated after a long (50%) or short (54%) interestrus interval. These results demonstrate that most (76%) pasture-based dairy cows in our analysis had at least one automated estrus alert within 6 wk postpartum and that both parity and genetic merit for fertility traits influenced the postpartum interval to return to estrus and reproductive performance. Automated activity monitoring systems provide a useful tool for characterizing pre-breeding estrus phenotypes and identifying cows with delayed estrus or atypical estrous cycles to inform targeted reproductive management.

## Highlights

• The first automated estrus alert occurred on 34 - 16 days postpartum (mean - SD).
• Shorter calving to first estrus intervals in multiparous and high-fertility cows.
• Most cows had normal interestrus intervals (18-24 days).
• First-service P/AI was greater for cows inseminated on second or subsequent estrus.
• First-service P/AI was greater for cows inseminated after a normal interestrus interval.

## Graphical Abstract

This retrospective observational study had 3 objectives: (1) characterize postpartum estrus phenotypes in spring-calving, pasture-based dairy cows; (2) evaluate associations between cow-level factors and the timing of return to estrus after calving; and (3) evaluate associations between pre-breeding estrus phenotypes and first-service pregnancy per artificial insemination (P/AI). The analysis included data from 4,586 lactations of pasture-based cows monitored using a neck-mounted activity system (SenseHub, MSD Animal Health). The first automated estrus alert occurred at 34 - 16 days postpartum (mean - standard deviation [SD]), with 25%, 51%, and 24% of cows classified as early (=21 days), intermediate (22-42 days), or late (>42 days) return to estrus after calving, respectively. Most interestrus intervals were of normal duration (18-24 days), whereas short (8-17 days) and prolonged (>24 days) intervals were more common after the first estrus than in later cycles.

**Figure:**
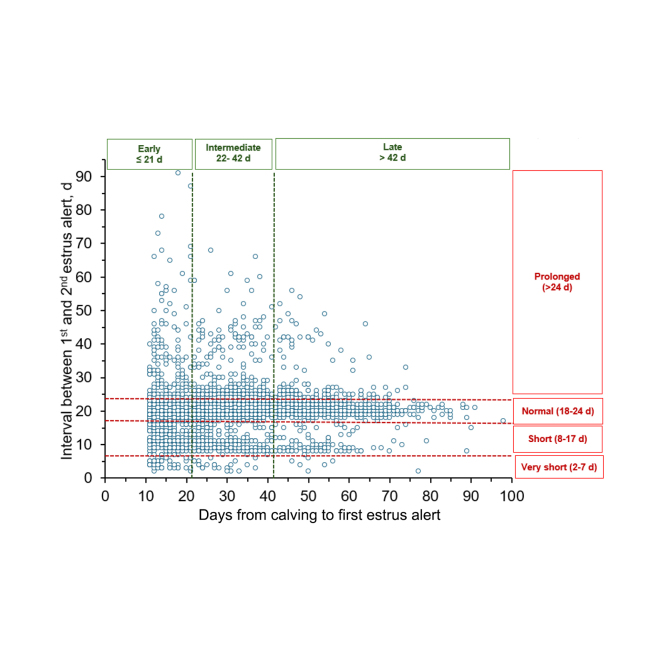

Herd reproductive performance is a key driver of the profitability and sustainability of seasonal-calving, pasture-based systems. High submission and conception rates are critical for maintaining a compact calving interval, which maximizes pasture utilization, improves labor efficiency, reduces replacement heifer requirements, and optimizes milk production per hectare (Shalloo et al., 2014). Failure to achieve timely conception, in turn, extends calving intervals, reduces herd productivity, and compromises overall farm efficiency.

The early postpartum period is a critical window when cows must achieve uterine involution and resume estrous cycles before a successful pregnancy can be established. In lactating dairy cows, regardless of calving system (e.g., year-round calving confinement systems or seasonal-calving, pasture-based systems), timely resumption of estrus expression and ovulation is essential to optimize reproductive performance (Walsh et al., 2007; Herlihy et al., 2013; Bruinjé et al., 2017). However, much of the foundational knowledge on postpartum ovarian dynamics and estrus expression comes from studies conducted in high-producing confinement systems, with fewer studies specifically examining these dynamics in spring-calving, pasture-based cows. The first ovulation usually occurs between 14 and 35 d after calving (Beam and Butler 1998; de Feu et al., 2009), but this initial ovulation is often “silent,” with no behavioral signs of estrus. The likelihood of behavioral estrus increases with each successive postpartum ovulation, likely reflecting prior exposure to progesterone during the preceding cycle, a longer interval from parturition and early lactation negative energy balance, and improved metabolic status (Kyle et al., 1992). The interval to first ovulation and estrus varies widely between cows and is influenced by factors such as parity, uterine health, bioenergetic status, body condition score, milk yield, environmental conditions, and genetics (Walsh et al., 2007; Moore et al., 2014). Delays in either the return to cyclicity or the expression of estrus have been consistently associated with reduced pregnancy per artificial insemination (**P/AI**), longer intervals from calving to conception, and an increased risk of culling (Dubuc et al., 2012; Borchardt et al., 2021).

Atypical estrous cycle patterns are increasingly recognized as indicators of underlying disruptions in ovarian or uterine physiology that compromise reproductive performance (Lucy, 2019). The first postpartum estrous cycle is often short, which is considered normal, but subsequent cycles generally occur within the characteristic 18–24 d interval. Nevertheless, more than 25% of postpartum cycles have been characterized as atypical or irregular, exhibiting patterns such as extended intervals to first ovulation, extended follicular phases, or prolonged luteal phases (Lamming and Darwash, 1998; Nyman et al., 2014; Bruinjé et al., 2025). These disruptions reflect deviations from normal ovarian function and often signal underlying problems with ovarian or uterine health, including premature luteolysis, anovulation, delayed luteolysis, cystic ovarian disease, or endometritis and pyometra (Lamming and Darwash, 1998; Bruinjé et al., 2024). Consequently, cows with atypical estrous cycles are more difficult to manage because estrus occurs at irregular intervals, and if they are inseminated, likelihood of conception is often reduced compared with cows displaying normal estrous cycles (Petersson et al., 2006; Bruinjé et al., 2017). The likelihood of cows displaying atypical cycles is influenced not only by physiological factors, but also by genetics; cows with poor genetic merit for fertility traits are more likely to exhibit abnormal cycle patterns, which contribute to reduced reproductive performance (Cummins et al., 2012b; Sitko et al., 2024). The prevalence of atypical cycles and associations with compromised reproductive performance highlight the need for postpartum monitoring.

Knowledge of the timing and patterns of estrus expression during the interval from parturition to the start of the breeding period is therefore essential for optimal herd reproductive management. Despite this, studies examining the relationship between estrus patterns and reproductive success in spring-calving, pasture-based dairy systems are limited. Therefore, this retrospective observational study had 3 objectives: (1) to characterize estrus phenotypes during the postpartum period in spring-calving, pasture-based dairy cows; (2) to evaluate associations between cow-level factors and return to estrus postpartum; and (3) to evaluate associations between pre-breeding estrus phenotypes and first-service P/AI. Insights from this work will support targeted reproductive management strategies for seasonal, pasture-based systems.

Data were available for 5,423 pasture-based lactating dairy cows across 24 herds in 2022 and 2023, totaling 8,859 lactation records. The herds were located across a broad geographic range in Ireland, primarily covering the Munster, Leinster, and Connacht regions. The dataset included calving records, genomic proofs, insemination and pregnancy diagnosis records, milk production, and automated estrus alerts (**AEA**) generated by automated activity monitoring (**AAM**) systems. Cows were predominantly Holstein-Friesian (85%) or crossbred (14%), with a small proportion of purebred Jersey (1%). The average herd size was 146 (minimum–maximum: 67–280) lactating cows.

Individual cow records were removed from analysis if specific exclusion criteria were met: (1) calving occurred outside the spring window (December to May) or after the herd mating start date (n = 242); (2) first artificial insemination (**AI**) occurred outside the spring window (April to Jully) or AI records were not available (n = 1,042); (3) the herd's reproductive records were incomplete (n = 183); (4) calving occurred within 42 d of the herd breeding start date (n = 1,139); (5) the AAM system collar was either not assigned by the calving date or estrus alert data were unavailable (n = 938); (6) the herd may have used estrus synchronization based on the observed frequency of AI events per day during the breeding season (n = 694); or (7) genetic merit for fertility subindex values (n = 30) or milk yield records (n = 5) were missing. The final dataset contained records for 2,984 pasture-based lactating dairy cows across 19 herds in 2022 and 2023, totaling 4,586 lactation records.

All cows were monitored with a neck-mounted activity monitoring device (SenseHub, MSD Animal Health). Detection of estrus during the mating season was conducted using standard reproductive management protocols within each farm and primarily relied on AEA to identify cows for insemination. Individual cow data and records of reproduction events were obtained from the Irish Cattle Breeding Federation database for each participating herd. The breeding start date (**BSD**) was defined separately for each herd-year as the first day of AI during the spring mating period. Conception status after the first insemination was determined using subsequent insemination event records within the same lactation, pregnancy diagnosis outcomes, and subsequent calving records.

The fertility subindex (**FI**) of the Economic Breeding Index reflects an animal's genetic merit for fertility and is calculated based on PTA values for calving interval and survival. The FI values for each cow were sourced from either March 2022 or March 2023 genetic evaluations, corresponding to the lactation year being analyzed. Cows were categorized into tertiles based on FI values (high [>€94.96], medium [€70.25–€94.96], low [<€70.25]). Daily milk production for each cow was estimated with the MilkBot model (Ehrlich, 2011) using milk test data retrieved from the Irish Cattle Breeding Federation database for each participating herd. Estimated cumulative milk yield up to 21 DIM (**Milk21**; high [>544.0 kg], medium [446.6–544.0 kg], low [<447.6 kg]) or milk yield at 80 DIM (**Milk80**; high [>30.8 kg/d], medium [25.8–30.8 kg/d], low [<25.8 kg/d]) were calculated for each cow. Cows were grouped according to days from calving to the first AEA (**FE** group: early [≤21 d], intermediate [22–42 d], late [>42 d]). Intervals between AEA were classified by cycle type (interestrus interval [**IEI**] type: very short [2–7 d], short [8–17 d], normal [18–24 d], prolonged [>24 d]). All IEI evaluated occurred before the first insemination.

All statistical analyses were conducted using SAS software (version 9.4; SAS Institute Inc.). First-service pregnancy per AI were analyzed using logistic regression via the GLIMMIX procedure in SAS. The interval from parturition to AEA was analyzed using Cox proportional hazard regression models with PROC PHREG in SAS, and survival curves were obtained using the Kaplan–Meier survival analysis of the MedCalc Software (version 23.2.8, MedCalc Software Ltd.). All models included the random effect of herd and fixed effect of parity (1 [n = 885], 2 [n = 1,022], 3 [n = 860], 4+ [n = 1,819]). Covariates offered to hazard of first AEA models included tertiles of FI (high [n = 1,513], medium [n = 1,561], low [n = 1,512]) and tertiles of Milk21 (high [n = 1,515], medium [n = 1,564], low [n = 1,507]). Cows classified as having a very short IEI after the first AEA (IEI 2–7 d; n = 89) were excluded from subsequent analyses of first-service P/AI. Covariates offered to first-service P/AI models, in addition to those previously mentioned, included prior postpartum AEA recorded before first AI (yes [n = 4,332], no [n = 165]), FE group (early [n = 1,142], intermediate [n = 2,355], and late [n = 1,089]), IEI type preceding first AI (short [n = 392], normal [n = 3,343], and prolonged [n = 477]), semen type (conventional [n = 3,635] vs. sex-sorted [n = 862]) and DIM at insemination. Milk80 (high [n = 1,466], medium [n = 1,539], low [n = 1,492]) was used in first-service P/AI models instead of Milk21. Multicollinearity was assessed using variance inflation factors (**VIF**), with all predictors considered acceptable with a VIF value <2. Manual backward elimination of covariates with *P* > 0.10 was adopted to select final models. Variables were considered significant if *P* ≤ 0.05, whereas *P*-values >0.05 and ≤0.10 were considered a tendency. When appropriate, the LSD post hoc mean separation test was used to determine differences between LSM. Unless otherwise stated, all reported proportions were calculated using the LSMEANS statement of GLIMMIX in SAS.

On average the first AEA occurred at 34 ± 16 d (mean ± SD) after calving. The distribution of cows across FE groups (first AEA <21 d, 22–42 d, or >42 d), was 25%, 51%, and 24%, respectively. The association between the interval from parturition to first AEA and the interval to the subsequent AEA is presented in Figure 1A, and the distribution of IEI is presented in Figure 1B. All intervals evaluated occurred before the first insemination. Across all IEI, normal IEI were predominant (79%), followed by short (10%), prolonged (9%), and very short (2%). Short and prolonged IEI were more prevalent after the first estrus (18% and 12%, respectively) compared with subsequent estrus events (4% and 8%, respectively).

**Figure 1:**
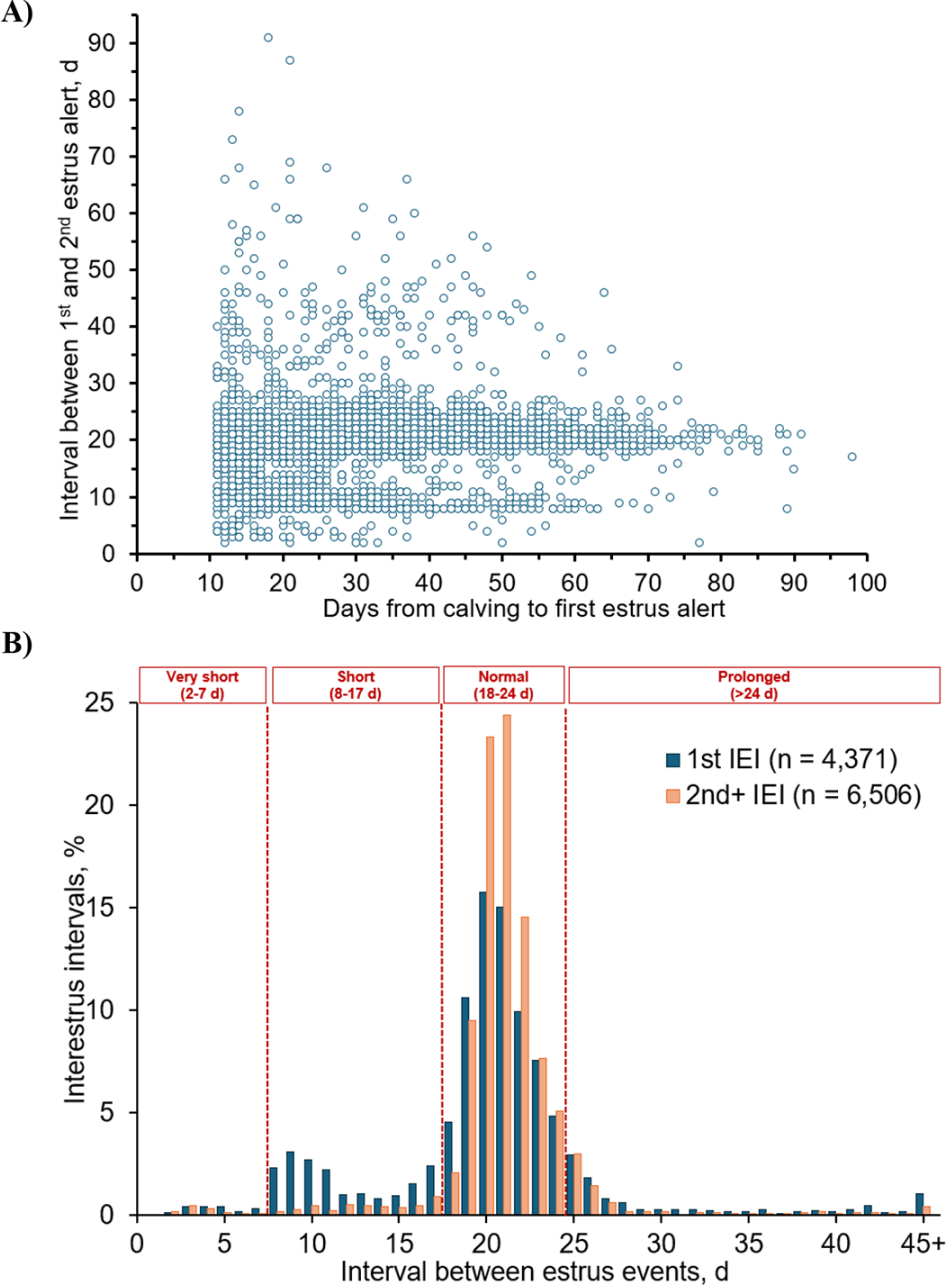
Estrus characteristics extracted from automated activity monitoring systems across 19 seasonal-calving, pasture-based herds. (A) Association between days from calving to the first automated estrus alert and the interval to the subsequent estrus alert (n = 4,586 lactations). (B) Distribution of the interval between automated estrus alerts for the first and subsequent IEI.

Survival curves for the interval from parturition to first AEA are presented in Figure 2. The hazard of estrus was lowest (*P* < 0.01) for parity 1 compared with other parity groups. Using parity 1 as the referent, parity 2 (hazard ratio [**HR**] = 1.41; 95% CI: 1.26–1.58), parity 3 (HR = 1.37; 95% CI: 1.22–1.55), and parity ≥4 (HR = 1.28; 95% CI: 1.13–1.44) had greater hazard of estrus. Using parity ≥4 as the referent, the hazard of estrus was greater for parity 2 (HR = 1.11; 95% CI: 1.01–1.21) but similar for parity 3 (HR = 1.08; 95% CI: 0.99–1.17). No significant differences were observed between parity 2 and parity 3 (HR = 1.03; 95% CI: 0.93–1.13). The hazard of estrus was lowest (*P* < 0.01) for the low FI tertile compared with the medium and high FI tertiles. Using the low FI tertile as the referent, the high FI tertile (HR = 1.14; 95% CI: 1.05–1.23) and the medium FI tertile (HR = 1.10; 95% CI: 1.02–1.18) had greater hazard of estrus. No significant differences were observed between the medium and high FI tertiles (HR = 1.04; 95% CI: 0.96–1.12). The hazard of estrus did not differ among Milk21 tertiles (*P* = 0.82).

**Figure 2:**
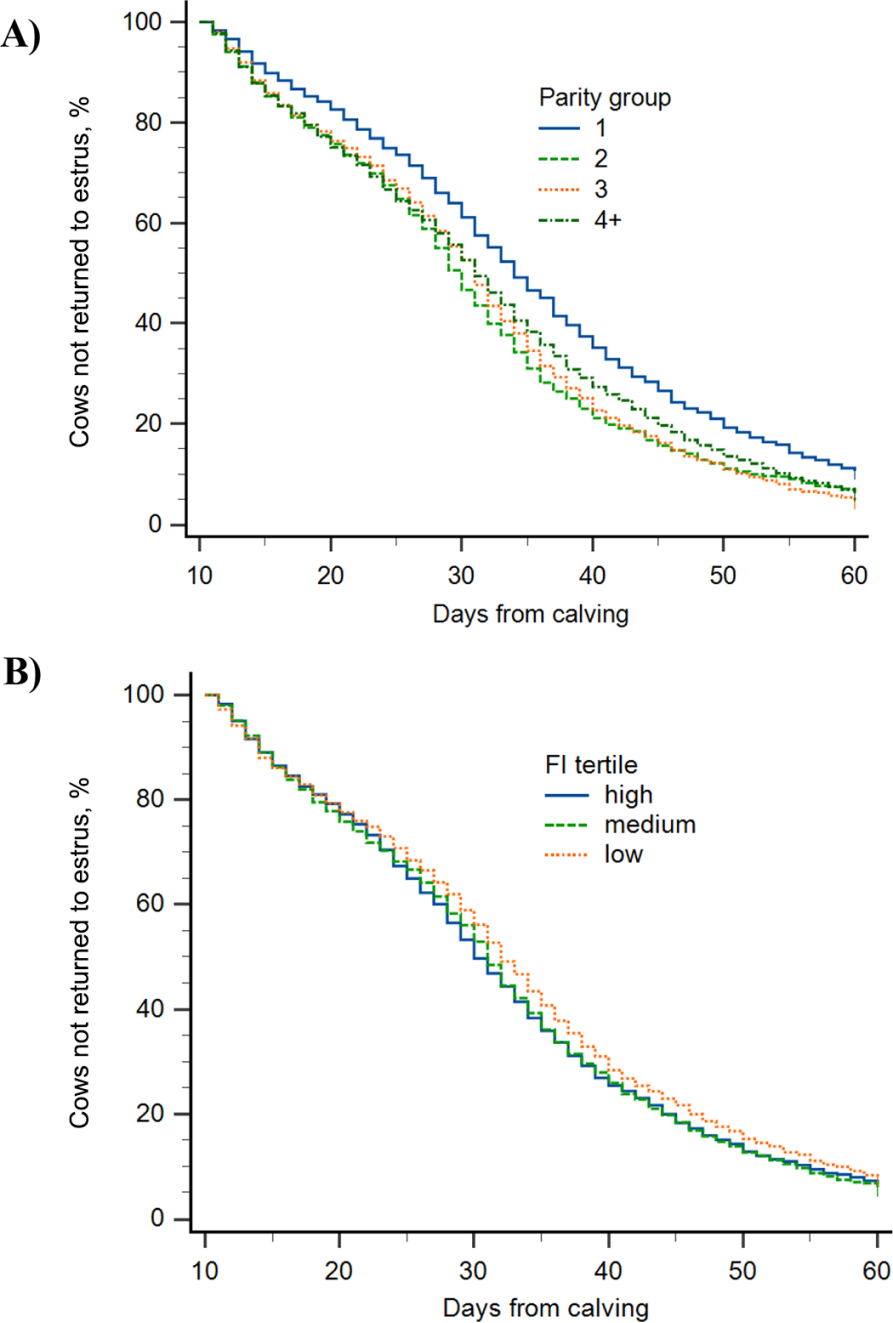
Survival curves for return to first estrus postpartum extracted from automated activity monitoring systems in 19 seasonal-calving, pasture-based herds (n = 4,586 lactations). Curves show the proportion of cows not yet returned to estrus on each day postpartum. (A) By parity (*P* < 0.01; 1 [n = 885], 2 [n = 1,022], 3 [n = 860], ≥4 [n = 1,819]). Median days to estrus were 34 d for parity 1, 30 d for parity 2, and 31 d for parity 3 and parity ≥4. (B) By fertility subindex (FI) tertile (*P* < 0.01; high [>€94.96; n = 1,513], medium [€70.25–€94.96; n = 1,561], low [<€70.25; n = 1,512]). Median days to estrus were 30 d for the high FI tertile, 31 d for the medium FI tertile, and 32 d for the low FI tertile.

The LSM and odds ratios from multivariable logistic regression models evaluating associations between cow-level variables and first-service P/AI are presented in Table 1. Pregnancy per AI at first service was greater (*P* = 0.02) for cows that had at least one postpartum AEA before first service compared with cows inseminated at the first detected estrus. Cows inseminated with conventional semen had greater (*P* = 0.02) first-service P/AI than those receiving sex-sorted semen. First-service P/AI tended (*P* = 0.10) to be greater for cows in high and medium FI tertiles than cows in the low FI tertile. Pregnancy per AI did not differ by parity (*P* = 0.33), FE group (*P* = 0.27), or Milk80 group (*P* = 0.67). To evaluate the association between IEI type and first-service P/AI, only cows with at least one previous AEA before first AI were included in the additional analysis (Table 1). Cows inseminated following a normal IEI had greater (*P* < 0.01) first-service P/AI compared with cows inseminated after a long IEI; cows inseminated after a short IEI were intermediate.

**Table 1:** Least squares means and odds ratio (OR) for first-service pregnancy per AI (P/AI) from multivariable logistic regression models; data from 19 seasonal-calving, pasture-based herds (n = 4,586 lactations)

Predictor^1^	First-service P/AI (n = 4,586)			First-service P/AI (only cows with prior AEA; n = 4,212)		
	LSM (95% CI)	OR (95% CI)	*P*-value	LSM (95% CI)	OR (95% CI)	*P*-value
FI			0.10			0.13
High	53.0 (47.5–58.4)	1.15 (0.98–1.34)		55.5 (51.3–59.6)	1.15 (0.98–1.36)	
Medium	53.3 (47.9–58.7)	1.16 (1.00–1.35)		55.3 (51.2–59.4)	1.15 (0.98–1.34)	
Low	49.6 (44.3–54.9)	Referent		51.9 (47.7–56.1)	Referent	
Parity			0.33			0.38
1	52.5 (46.7–58.2)	Referent		54.6 (49.8–59.2)	Referent	
2	51.0 (45.3–56.6)	0.94 (0.78–1.14)		52.8 (48.3–57.2)	0.93 (0.77–1.13)	
3	54.0 (48.3–59.8)	1.07 (0.88–1.29)		56.5 (51.7–61.1)	1.08 (0.88–1.32)	
4+	50.4 (45.1–55.7)	0.92 (0.78–1.09)		53.2 (49.1–57.2)	0.94 (0.79–1.13)	
Prior AEA			0.02			—
Yes	56.6 (53.4–59.7)	Referent		—	—	
No	47.4 (39.1–55.8)	0.69 (0.50–0.95)		—	—	
IEI type	—	—	—			<0.01
Short (2–7 d)	—	—		54.4 (48.6–60.0)^ab^	0.86 (0.69–1.07)	
Normal (18–24 d)	—	—		58.1 (55.0–61.1)^a^	Referent	
Prolonged (>24 d)	—	—		50.3 (44.9–55.5)^b^	0.73 (0.60–0.88)	
Semen type			0.02			0.01
Conventional	54.6 (49.8–59.2)	Referent		57.1 (53.8–60.4)	Referent	
Sex-sorted	49.4 (43.5–55.4)	0.81 (0.69–0.96)		51.3 (46.6–56.1)	0.79 (0.67–0.94)	
DIM at AI	—	—	<0.01	—	—	<0.01
FE group	—	—	0.27	—	—	0.90
Milk80 group	—	—	0.67	—	—	0.34

a,b Different superscripts indicate significant differences (*P* ≤ 0.05) between groups.

1 FI = fertility subindex; AEA = automated estrus alert; IEI = interestrus interval; FE = first AEA.

This retrospective observational study characterized estrus phenotypes during the postpartum pre-breeding period in spring-calving, pasture-based dairy cows. We described return to estrus after calving and subsequent IEI patterns, evaluated associations between cow-level factors and timing of return to estrus, and examined relationships between pre-breeding estrus phenotypes and first-service P/AI. These findings contribute to a better understanding of the reproductive status of lactating dairy cows in seasonal, pasture-based production systems.

In seasonal breeding systems, cows not detected in estrus by the start of the breeding period are either truly anovulatory anestrus, ovulatory without detected estrus (often only 10%–20%), or calved late and not yet had sufficient time to resume cyclicity. In the present study, the average interval from calving to the first AEA was 34 ± 16 d. Consistent with studies that evaluated return to cyclicity using progesterone profiles (Santos et al., 2009; Nyman et al., 2014), primiparous cows had a lower hazard of first estrus and a greater proportion were anestrous at 42 DIM than multiparous cows. The longer postpartum anestrous interval in primiparous cows likely reflects the combined challenges of continued growth, high metabolic demands, and greater susceptibility to calving difficulties and uterine disease.

Overall, 76% of cows had at least one AEA by 6 wk postpartum and most cows (96%) had at least one AEA before the herd BSD (on average, cows were 73 ± 13 DIM at BSD) and only 4% did not, a very low proportion that partly reflects the exclusion of late-calving cows (<42 DIM at BSD) from this analysis. In a larger evaluation of the full population (Sitko et al., 2026), which retained all cows regardless of DIM at BSD, 11% of cows were anestrous at the BSD, with herd-level prevalence ranging from 5% to 32%, consistent with an earlier New Zealand report of 13% to 48% anovulatory cows at the onset of breeding (Rhodes et al., 2001). Collectively, these results underscore the role of calving distribution in influencing the proportion of anestrous cows at the herd BSD.

Early postpartum estrous cycles are inherently more variable than subsequent cycles. In the current study, the first IEI was more likely to be classified as atypical compared with subsequent IEI. These findings are consistent with Nyman et al. (2014), who also observed more atypical patterns in first estrous cycles, with 40% and 30% incidence of short and long cycles, respectively. It is well recognized that some cows exhibit short estrous cycles after the first postpartum ovulation: premature release of PGF_2α_ from the uterus often occurs, and the corpus luteum is more sensitive to luteolytic signals (Zollers et al. 1993; Rhodes et al., 2003). In subsequent cycles, the corpus luteum generally has sustained luteal function and a normal period of activity. Prolonged cycles after the first estrus may indicate a persistent corpus luteum, which can result from transient uterine health issues or other early lactation challenges that impair luteolytic mechanisms (Herath et al., 2009; Sheldon et al., 2009). Our findings contrast with Denis-Robichaud et al. (2024), who reported a much greater proportion of cows with long (20%; 24–30 d) or very long cycles (40%; >30 d), despite the use of hormonal synchronization. This difference is notable because long estrous cycles were distinguished from potential pregnancy loss in both studies: in the current study, the interestrus intervals evaluated occurred before first insemination, whereas Denis-Robichaud et al. (2024) included a noninseminated group. Consequently, the observed differences in the proportion of long cycles between the 2 studies may reflect differences in how cycle length was estimated or variations in management, environmental conditions, and genetics.

Among cows that had returned to estrus before the start of the breeding season, we observed an average reduction of 6 percentage points in first-service P/AI in cows inseminated at detected estrus following an atypical IEI (≤17 d or ≥25 d) compared with cows inseminated following a normal IEI (18–24 d). This concurs with Bruinjé et al. (2017), who reported that cows that had abnormal estrous cycle duration were associated with suboptimal ovarian or uterine function, which in turn was associated with reduced fertility. Short cycles are often associated with premature luteolysis, inadequate luteal function, or delayed ovulation, whereas long cycles may reflect poor estrus detection, silent ovulations, prolonged luteal phases, or extended proestrus. These abnormalities compromise fertility by disrupting luteal function, impairing endometrial receptivity, reducing oocyte quality, or causing suboptimal timing of insemination relative to ovulation. In contrast, a normal IEI generally reflects a well-functioning ovarian and uterine environment and is associated with accurate estrus detection and optimal fertility.

Genetic merit for fertility traits also influenced reproductive outcomes. The interval from calving to first estrus was shorter and first-service P/AI was greater for cows with medium or high FI, underlining the important role of selecting for cow FI to improve reproductive performance. These results are consistent with previous studies that compared cows with divergent genetic merit for fertility traits (Cummins et al., 2012a,b; Moore et al., 2014); cows with superior genetic merit for fertility had earlier postpartum resumption of estrous cyclicity, greater luteal phase progesterone concentrations, stronger behavioral estrus and greater P/AI. Together, these studies indicate that genetic merit for fertility traits affects both estrous cycle phenotypes and reproductive performance.

Automated activity monitors are valuable for large-scale estrus surveillance, offering continuous, noninvasive detection of estrus-related activity changes. Nonetheless, their usefulness for accurately capturing cyclicity information is variable. Borchardt et al. (2025) reported a high specificity but only moderate sensitivity for detecting cyclicity, indicating that some cows that had resumed ovarian activity were not identified. This limitation is partly due to the high proportion of first postpartum ovulations that are silent. Therefore, our estimates of time to first estrus may represent a conservative measure of true cyclicity. Nonetheless, cows that had at least one AEA before first service achieved greater P/AI than cows inseminated at their first detected estrus postpartum (57% vs. 47%). Notably, if the cow had at least one AEA during the pre-breeding period, the exact timing of the first estrus relative to calving (i.e., ≤21 d, 22–42 d, or ≥42 d) was not associated with first-service P/AI.

These findings, together with previous work (Borchardt et al., 2021; Rial and Giordano, 2024), underscore the value of AAM systems to identify anestrous cows or cows with atypical cycle patterns before BSD, thereby enabling targeted interventions and more strategic semen allocation. Leveraging pre-breeding information (e.g., data on AEA, genetic merit, parity, health) to identify cows with lower fertility potential in advance allows for proactive interventions, such as synchronization of ovulation and timed AI, to improve both submission and conception rates. Accordingly, cows without an AEA by BSD, regardless of calving date, are potential candidates for hormonal synchronization and timed AI, with earlier-calving cows typically treated at BSD and later-calving cows receiving intervention a few weeks into the breeding season when they become eligible. Notably, the absence of a pre-breeding AEA should be viewed as a screening indicator rather than a definitive diagnosis; follow-up examination (e.g., assessment of corpus luteum status) is required to distinguish truly anovulatory cows from those that are cycling but not detected and to guide appropriate intervention strategies. Further, although genetic merit traits remain the primary criteria for selecting replacement heifers, AEA status and interestrus patterns may provide complementary information on the likelihood of conception and thus help to refine semen allocation strategies, with sex-sorted semen prioritized for cows of higher probability of conception and beef semen directed toward cows with lower probability of conception. Such a strategy has the potential to enhance genetic gain in replacement heifers, increase calf crop value, and support dairy-beef integration, thereby aligning reproductive decisions with broader goals related to labor efficiency and whole-farm sustainability, objectives that are particularly important in seasonal, pasture-based systems where condensed calving periods present unique challenges.

Collectively, our data highlight that seasonal-calving, pasture-based cows exhibit timely postpartum return to estrus and maintain largely normal postpartum estrous cycles. Notably, the high proportion of cows with at least one AEA by herd BSD was likely influenced, in part, by the exclusion of cows calved less than 42 d before BSD. Although excluding late-calving cows minimized the confounding effect of insufficient time to express estrus, it may also have omitted a subset of cows more prone to delayed estrus or atypical cycle patterns. In addition, not all cows in the original dataset had complete AEA or AI records, which limited the number of cows available for analysis. Nonetheless, delayed return to estrus and atypical IEI were associated with reduced first-service P/AI, underscoring their relevance for reproductive success in seasonal systems with a short breeding period. Monitoring estrus phenotypes through automated activity systems represents a promising strategy to enhance fertility outcomes by supporting targeted management interventions. Although we identified some risk factors for delayed return to estrus, we did not have access to other sources of information that would have improved this capacity (e.g., clinical health records). Future work should focus on improving data capture and integration across herd management systems, as well as identifying risk factors for cows diagnosed as anestrous or with atypical cycles and refining targeted intervention strategies for these cows.
